# Monitoring COVID-19 Transmission Risks by Quantitative Real-Time PCR Tracing of Droplets in Hospital and Living Environments

**DOI:** 10.1128/mSphere.01070-20

**Published:** 2021-01-06

**Authors:** Andrea Piana, Maria Eugenia Colucci, Federica Valeriani, Adriano Marcolongo, Giovanni Sotgiu, Cesira Pasquarella, Lory Marika Margarucci, Andrea Petrucca, Gianluca Gianfranceschi, Sergio Babudieri, Pietro Vitali, Giuseppe D’Ermo, Assunta Bizzarro, Flavio De Maio, Matteo Vitali, Antonio Azara, Ferdinando Romano, Maurizio Simmaco, Vincenzo Romano Spica

**Affiliations:** a Department of Medical, Surgical and Experimental Sciences, University of Sassari, Sassari, Italy; b Department of Medicine and Surgery, University of Parma, Parma, Italy; c Department of Movement, Human and Health Sciences, Laboratory of Epidemiology and Biotechnologies, University of Rome “Foro Italico,” Rome, Italy; d Sant’Andrea Hospital, Sapienza University of Rome, Rome, Italy; e Department of Surgery “P. Valdoni”, Sapienza University of Rome, Rome, Italy; f Department of Basic Biotechnological Sciences, Intensive and Perioperative Clinics, Section of Microbiology, Catholic University of the Sacred Heart, Rome, Italy; g Department of Public Health and Infectious Diseases, University of Rome La Sapienza, Rome, Italy; Mount Sinai School of Medicine

**Keywords:** SARS-CoV-2, biological fluids, droplets, environmental contamination, fomite, microbiota

## Abstract

Severe acute respiratory syndrome coronavirus 2 (SARS-CoV-2) environmental contamination occurs through droplets and biological fluids released in the surroundings from patients or asymptomatic carriers. Surfaces and objects contaminated by saliva or nose secretions represent a risk for indirect transmission of coronavirus disease 2019 (COVID-19). We assayed surfaces from hospital and living spaces to identify the presence of viral RNA and the spread of fomites in the environment. Anthropic contamination by droplets and biological fluids was monitored by detecting the microbiota signature using multiplex quantitative real-time PCR (qPCR) on selected species and massive sequencing on 16S amplicons. A total of 92 samples (flocked swabs) were collected from critical areas during the pandemic, including indoor (three hospitals and three public buildings) and outdoor surfaces exposed to anthropic contamination (handles and handrails, playgrounds). Traces of biological fluids were frequently detected in spaces open to the public and on objects that are touched with the hands (>80%). However, viral RNA was not detected in hospital wards or other indoor and outdoor surfaces either in the air system of a COVID hospital but only in the surroundings of an infected patient, in consistent association with droplet traces and fomites. Handled objects accumulated the highest level of multiple contaminations by saliva, nose secretions, and fecal traces, further supporting the priority role of handwashing in prevention. In conclusion, anthropic contamination by droplets and biological fluids is widespread in spaces open to the public and can be traced by qPCR. Monitoring fomites can support evaluation of indirect transmission risks for coronavirus or other flu-like viruses in the environment.

**IMPORTANCE** Several studies have evaluated the presence of SARS-CoV-2 in the environment. Saliva and nasopharyngeal droplets can land on objects and surfaces, creating fomites. A suitable indicator would allow the detection of droplets or biofluids carrying the virus. Therefore, we searched for viral RNA and droplets and fomites on at risk surfaces. We monitored by qPCR or next generation sequencing (NGS) droplets through their microbiota. Although the study was performed during the pandemic, SARS-CoV-2 was not significantly found on surfaces, with the only exception of environmental areas near infectious patients. Conversely, anthropic contamination was frequent, suggesting a role for biofluids as putative markers of indirect transmission and risk assessment. Moreover, all SARS-CoV-2-contaminated surfaces showed droplets’ microbiota. Fomite monitoring by qPCR may have an impact on public health strategies, supporting prevention of indirect transmission similarly to what is done for other communicable diseases (e.g., influenza and influenza-like infections).

## Abstract (precis)

Several studies evaluated the presence of SARS-CoV-2 in the environment. Saliva and nasopharyngeal droplets can land on objects and surfaces, creating fomites.

## INTRODUCTION

The ongoing pandemic of coronavirus disease 2019 (COVID-19) epidemiologically depends on person-to-person close contacts and inhalation of virus-laden liquid droplets (1–6). However, it is suggested that severe acute respiratory syndrome coronavirus 2 (SARS-CoV-2) could be indirectly transmitted through environmental contamination of objects and surfaces by biological fluids (e.g., saliva, nose secretion, urine, or fecal samples) (7–16). Respiratory droplets (aerodynamic diameter ranging between 6 and 10 μm) and droplet nuclei or aerosols (≤5 μm) can directly reach the mouth, nose, or eyes of a susceptible person but can also land on surfaces (17–20). Coronaviruses can survive on different matrices under different conditions, persisting from hours to days, especially in indoor environments (21–23).

Knowledge about environmental contamination is important during outbreaks and transition phases to enforce public health measures for symptomatic and asymptomatic individuals (22–24). Concerns about environmental contamination and the associated risk of indirect transmission can be raised in environments at highest risks (e.g., hospitals), where SARS-CoV-2 was detected, even when sanitation was accurately performed (8, 9, 13, 24–29).

**FIG 1:**
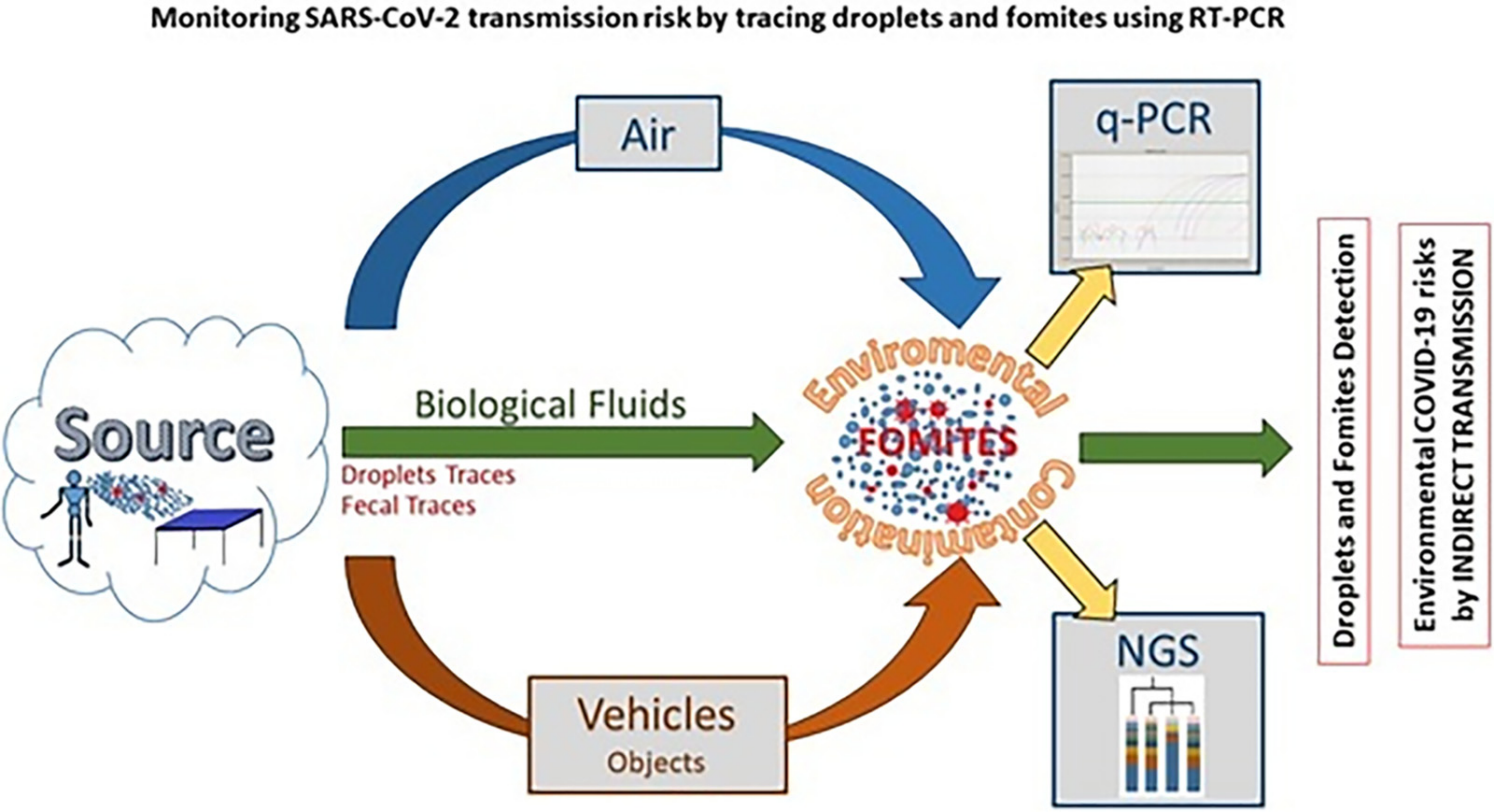
Schematic representation of aim and focus of this study. The aim and focus of this study were to search for both SARS-CoV-2 and fomites in hospitals and public buildings in order to evaluate qPCR monitoring of fomites and biofluids as indicators of hygiene as well as candidate markers of COVID-19 transmission through an indirect route of infection.

The detection of fomites and biological fluids in the environment can be used as a potential marker of hygiene and of indirect SARS-CoV-2 transmission. On this basis, hospitals and public buildings were surveyed for the presence of SARS-CoV-2, droplets, fomites, and anthropic contaminations: traces of the microbiota signature of their own biofluids of origin were searched. A dedicated set of primers and probes was combined to detect different biological fluids based on multiplex reactions in quantitative real-time PCR (qPCR), following a strategy initially developed for forensic studies and hospital hygiene (30–34). A real-time PCR-based approach was adopted to test SARS-CoV-2 RNA (reverse transcriptase quantitative PCR [RT-qPCR]) and bacterial DNA from fomites by qPCR microbiota signature, amplifying genes from at least one representative bacterial component of the biological fluid (e.g., Streptococcus salivarius and Streptococcus mutans for saliva, *Corynebacterium* for nose secretions, Enterococcus faecalis and *Bacteroides* for fecal traces) (35–39). Findings were validated by next generation sequencing (NGS), which evaluated the microflora DNA (mfDNA) sampled with environmental swabs on indoor and outdoor surfaces (40–44). Although NGS is time-consuming and requires a bioinformatic analysis, it provides a larger view of the microbiota components, including the bacterial indicators selected for qPCR. qPCR and NGS can be used on the same samples to characterize the environmental microflora by cycle threshold (*C_T_*) analysis of selected marker genes or by read counts on 16S amplicon sequences, respectively (Fig. 1). High-throughput analysis of 16S rDNA by NGS can provide sequences of hundreds of bacterial species helping to define the whole microbiota, so that the anthropic contamination becomes a component of the whole microflora detectable on a surface. Both NGS and qPCR analyze DNA without providing information on the vitality of the biological agents. While NGS requires specific equipment and protocols, the qPCR approach uses the same instruments and procedures of the RT-qPCR.

The aim of this study was to assess the presence of SARS-CoV-2 and fomites in hospitals and public buildings in order to evaluate qPCR monitoring of fomites and biofluids as indicators of hygiene, as well as markers of SARS-CoV-2 transmission.

## RESULTS AND DISCUSSION

### Detection of fomites by qPCR is a feasible and effective approach.

**FIG 2:**
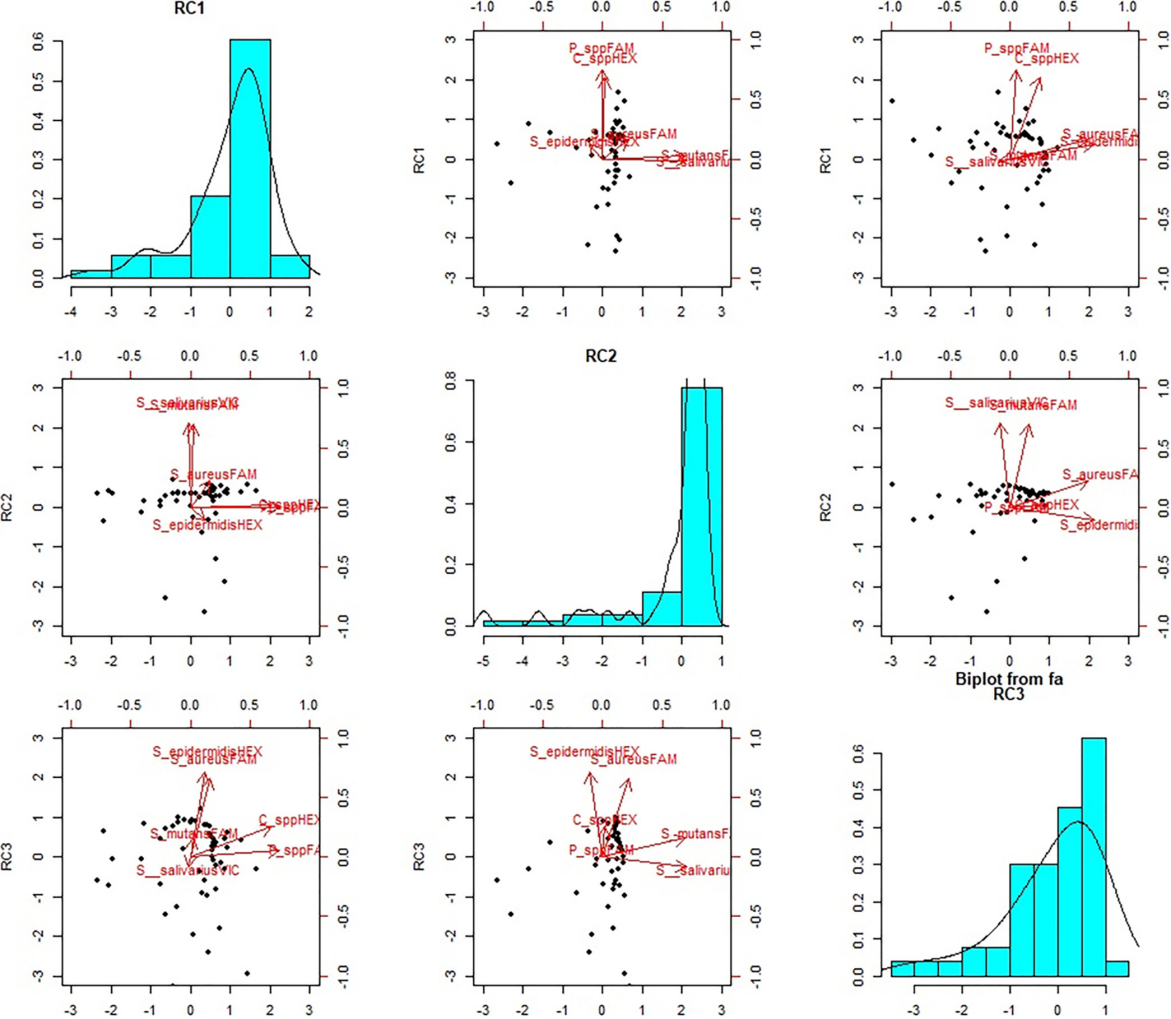
Droplet component distribution. Principal-component analysis biplots showing component 1 versus component 2, component 1 versus component 3, and component 2 versus component 3. The first, second, and third components explain 28%, 27%, and 27% of the variability observed, respectively. The role of the bacterial indicators within the different components is summarized by vectors within the scatter graphs.

Anthropic contamination by droplets and biofluids was detected on several environmental surfaces by qPCR (Table 1). Fomites were found in indoor and outdoor areas exposed to human crowding or frequently touched by hands. Floors and walls were less contaminated than handles or buttons. Droplet DNA traces were detected in the majority of the sampled surfaces, and almost 10% of sampled points displayed multiple contamination from different biological fluids. Correlation between selected bacterial species and biological fluids in droplets and fomites was confirmed (*P* value < 0.01) (Table 2), supporting the effectiveness of the approach. The combined action of different markers is synergic (Fig. 2), allowing a reliable identification of droplets and fomites. Indoor and outdoor samples showed the presence of traces from one or more human biofluids, although with different frequencies. qPCR can show not only the presence of biofluids and droplet traces but also the contamination level. Automated nucleic acid extraction and detection of anthropic contamination from environmental swabs could be implemented following the example adopted for the management of human swabs; therefore, detection of fomites by qPCR seems a feasible and promising approach even on a larger scale.

**TABLE 1:** Anthropic contamination by real-time PCR: cumulative results for each indicator^*a*^

	Code	Skin	Droplets		Feces	Sample description
Sample source			Nasopharynx	Oropharynx		
Controls (*n* = 4)	A_01	−	+++	−	−	Droplet biofluid
	A_02	++++	+++++	+	−	Droplet biofluid
	A_03	++++	+++++	++	−	Droplet biofluid
	A_04	++++	++++++	+	−	Droplet biofluid
Outdoor (*n* = 6)	Z_01	+++	+++	−	+	Handrail
	Z_02	+/−	+	−	−	Bus stop bench
	Z_03	++	++	−	+/−	Shared e-scouter grip
	Z_04	−	+	−	−	External door handle
	Z_05	++	+	−	+/−	External door handle
	Z_06	++	+	−	−	Coffee dispenser
Indoor (*n* = 55)	YH1_01	+++	++	+	+	Right bed rail*
	YH1_02	+++	++	+/−	+/−	Bedside table
	YH1_03	++	+++	++++	+++	Door handle
	YH1_04	++	+++	−	+/−	Floor
	YH1_05	++	++++	+	+++	Call button*
	YH1_06	++	++	−	−	Table
	YH1_07	−	++	−	−	Chair
	YH1_08	−	+++	−	−	Back of the bed
	YH1_09	−	++	−	−	Air inlet socket
	YH1_10	+++	++	+++	−	Wall behind the bed
	YH1_11	+++	+++	+++	++	Left bed rail
	YH1_12	−	+++	−	−	Stethoscope*
	YH1_13	−	+++	−	−	Bottom of the bed
	YH1_14	+	++++	−	+/−	Wheelchair head
	YH2_15	++++	++	−	−	Pillow
	YH2_16	−	+	−	−	Chair
	YH2_17	−	+	−	−	Back of the bed
	YH2_18	−	+/−	−	+/−	Toilet board
	YH2_19	−	+/−	−	+	Sink faucet
	YH2_20	−	++	−	−	Floor
	YH2_21	+	+/−	−	−	Floor
	YH2_22	−	++	++++	−	Floor
	YH2_23	−	+	−	−	Door handle
	YH2_24	+++	++	−	+/−	Back of the bed
	YH2_25	++	+	++++	−	Side of bed
	YH2_26	+	+	−	+++	Side of bed
	YH2_27	−	−	−	−	Bedside table
	YH2_28	−	+/−	−	+	Bedside table
	YH2_29	−	++	−	−	Pillow
	YH2_30	−	+	−	−	Bed sheets
	YH2_31	+	++	−	−	Floor
	YH2_32	−	++	−	−	Floor
	YH2_33	−	+	−	−	Floor
	YH3_34	++++++	++++	−	−	Toilet
	YH3_35	−	++	−	−	Toilet
	YH3_36	+/−	−	−	−	Door handle
	YC_37	−	+	−	−	Pew and surface
	YC_38	−	+	−	−	Pew and surface
	YC_39	−	+	−	−	Pew and surface
	YC_40	++	++++	+	−	Pew and surface
	YO_41	−	−	−	+	Floor
	YO_42	−	−	+/−	+/−	Floor
	YO_43	−	−	−	+	Floor
	YO_44	−	−	−	+	Floor
	YO_45	−	−	−	−	Floor
	YO_46	−	−	−	+	Floor
	YO_47	−	−	−	+/−	Toilet wall tiles
	YO_48	−	−	−	+	Toilet wall tiles
	YO_49	−	−	−	+	Toilet wall tiles
	YO_50	−	−	−	+/−	Office phone
	YO_51	−	−	−	+	Computer keyboard
	YO_52	+/−	−	−	+	Computer keyboard
	YO_53	+	+	+	+	Elevator handle
	YO_54	+++	+++	+	+/−	Toilet door handle
	YO_55	++	++	−	−	Toilet flush button

a The cumulative output of indicators for each anthropic contamination is shown, including the description of each sample. For each biological fluid, at least two indicators were used: +++, positive with *C_T_* < 20; ++, positive with *C_T_* of 21 to 30; +, positive with *C_T_* of 31 to 35; +/−, low-confidence positive with *C_T_* of 36 to 38; −, negative *C_T_* >39; for E. coli, ++, positive with *C_T_* < 20; +, positive with *C_T_* of 21 to 29; +/−, low-confidence positive with *C_T_* of 30 to 35; −, negative with *C_T_* > 36. The sampling points where SARS-CoV-2 RNA was detected are indicated by an asterisk. Sample reading code: A, anthropic; Z, outdoor; Y, indoor (H, hospital; C, church; O, office and restaurant).

**TABLE 2:** Primary indicators for droplets^*a*^

Origin	qPCR droplet markers			
	Indicators	*RC1*	*RC2*	*RC3*
Nasopharynx	*Propionibacterium* spp.	**0.93**	0.05	−0.01
	*Corynebacterium* spp.	**0.66**	−0.03	0.39
Oropharynx	Streptococcus mutans	0.21	**0.76**	0.11
	Streptococcus salivarius	0.02	**0.89**	−0.15
Nostril-skin	Staphylococcus aureus	0.06	0.30	**0.80**
	Staphylococcus epidermidis	0.13	−0.07	**0.88**

a Pattern matrix for principal-component analysis nasopharynx selected indicators (*Propionibacterium* spp., *Corynebacterium* spp.), oropharynx (Streptococcus mutans, *Staphylococcus salivarius*), of nostril-skin (Staphylococcus aureus, Staphylococcus epidermidis). The higher correlations for each component (RC) are shown in boldface type (*P* < 0.01). The table reported variable loading on the rotation matrix.

### SARS-CoV-2 on surfaces in hospitals and public places is not widely disseminated, unless in proximity of an infected patient.

SARS-CoV-2 was not detected in the majority of the indoor and outdoor sampling points, including all 15 sampling points within the air system of a COVID-19 hospital. SARS-CoV-2 RNA was detected only in one room where an infected patient was hospitalized and only in those samples collected near the patient (one on the bed rail and one on the surface of the call button). Furthermore, the stethoscope used for the patient was also positive. The lower (<4%) frequency of positive samples in comparison with other studies (20 to 30%) can be explained by the epidemiology rather than to differences in the sampling strategy or to a lower sensitivity of the method (36); other studies were conducted during the epidemic peak, whereas our study was carried out after the lockdown, when reopening of activities was carefully performed and preventive measures were strictly enforced (7–16, 18, 45–47). Environmental spread of SARS-CoV-2 was not relevant, with the only exception of the surfaces near a hospitalized infected patient.

### NGS confirms microbial signature by qPCR.

**FIG 3:**
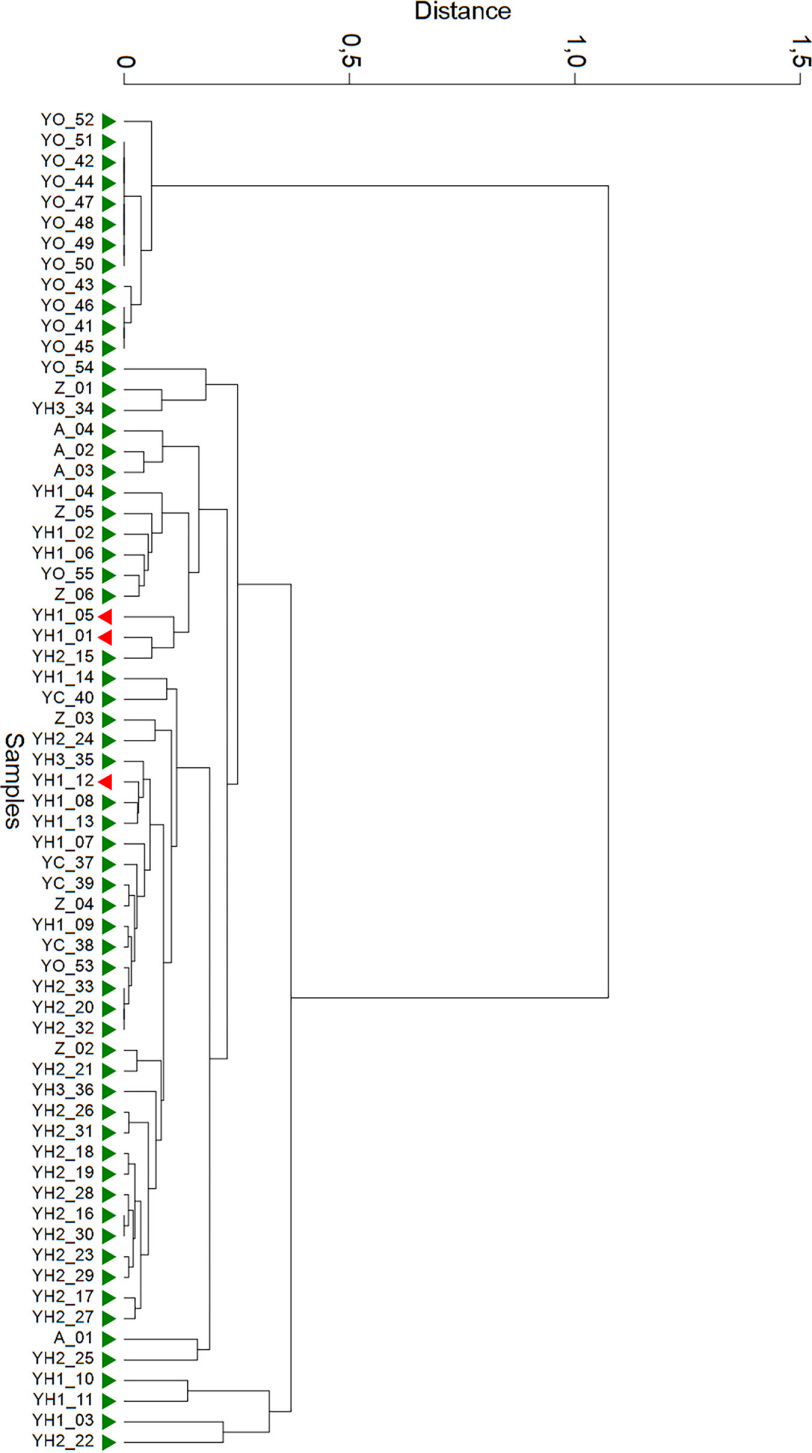
Hierarchical cluster analysis on real-time PCR results. SARS-CoV-2 positive and negative samples are indicated in green and red, respectively. The hierarchical cluster was performed on raw *C_T_* data by Euclidean distance and Ward’s linkage (clustering to minimize the sum of squares of any two clusters).

**FIG 4:**
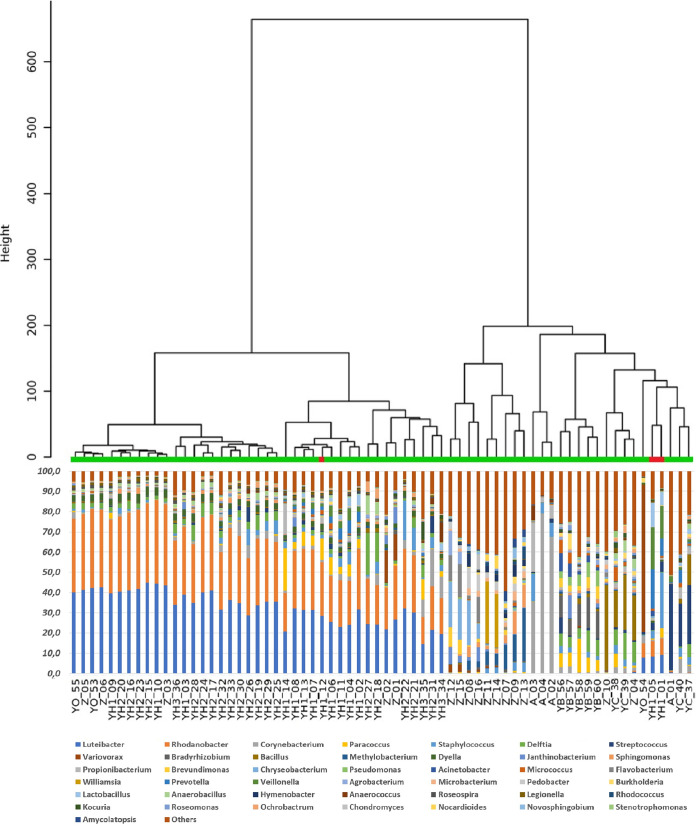
Hierarchical clustering dendrogram on 16S amplicon sequencing data. Dendrogram shows a hierarchical clustering of samples based on genus-level classifications. The bar chart under each sample summarizes the relative abundance of its genus-level classifications. In this analysis were included also environmental samples from playgrounds (Z_07-16) and indoor air (YB_56-60), without major anthropic contaminations. SARS-CoV-2-negative and -positive samples are indicated in green and red, respectively.

**FIG 5:**
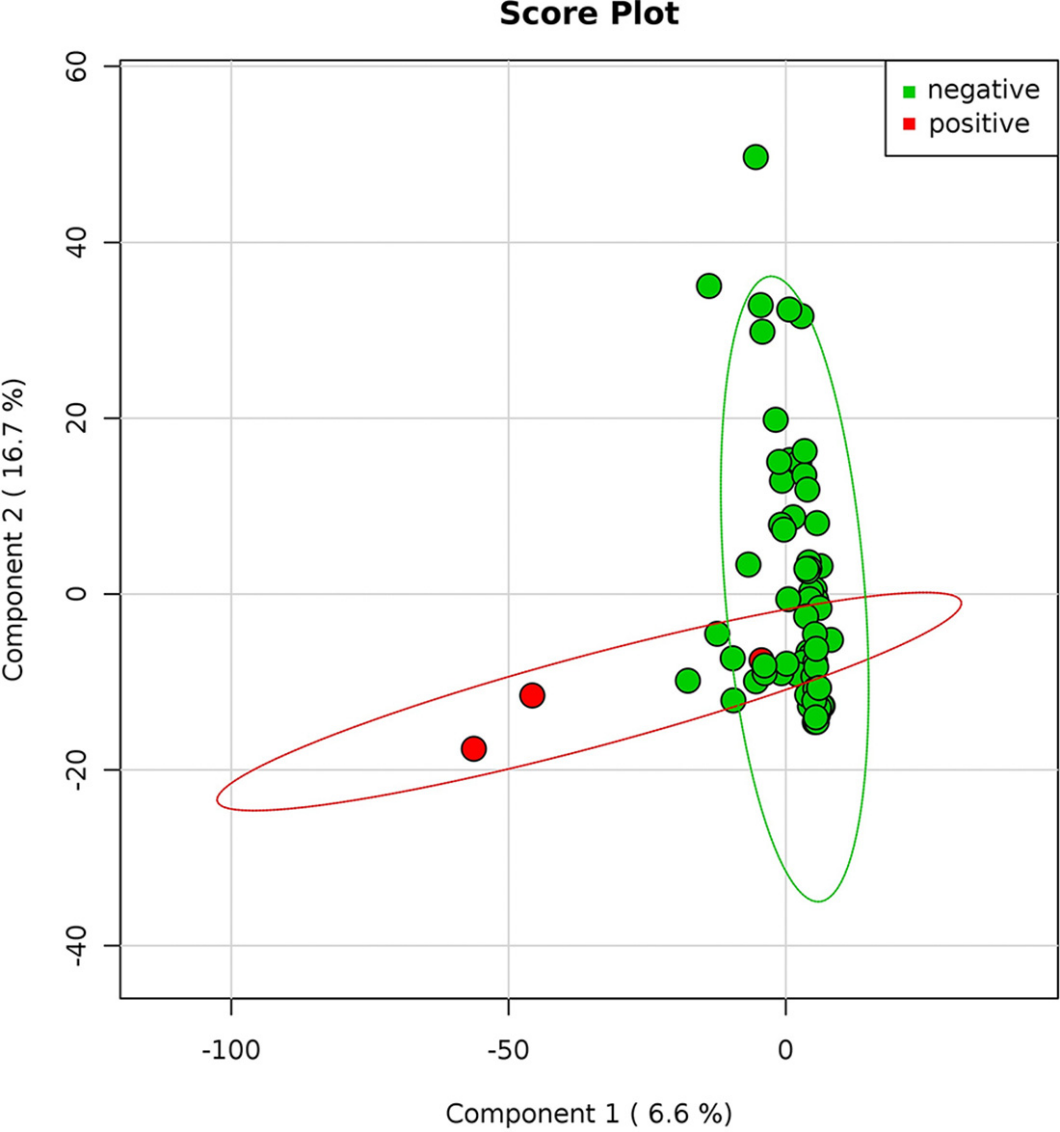
SARS-CoV-2-positive and -negative samples. Principal-coordinate analysis of the normalized relative abundance of all samples divided by negative and positive results of SARS-COV2. Data are plotted at the genus-level classification. The variance is explained for 6.6% and 16.7%, respectively for components 1 and 2. SARS-CoV-2 positive (red) and negative (green) samples are indicated.

The microbial signature was confirmed by NGS, and all selected bacterial indicators were included within the microbiota identified by high-throughput sequencing and bioinformatic analysis. Both qPCR and NGS showed the prevalent contamination patterns (Fig. 3 and 4). DNA test can be easily performed within 1 day on any real-time apparatus, whereas NGS can be performed within 1 week, adapting the laboratory protocol to the high-throughput sequencer. Therefore, each selected indicator amplified by qPCR was found by NGS, but only as a subcomponent between others (about 200 to 1,000 species for each sample), including unknown species (∼5 to 10%). Mean values ranged from 0.24% (*Bacteroides*) to 5.78% (*Corynebacterium*). For example, *Corynebacterium* showed lower values (<1%) in environmental samples exposed to multiple sources of microbial pollutions, whereas the highest values (35 to 80%) were observed for environmental swabs contaminated by nasopharyngeal secretions. The correspondence between qPCR and NGS was reliable: two independent SARS-CoV-2-positive samples (YH1_01 and YH1_05) of a patient surroundings closely gathered, whereas the sample collected from the stethoscope (YH1_12) segregated at a different distance (Fig. 5). The anthropic contaminations on the right side of the bed (YH1_01) and on the call bottom (YH1_05) showed a similar biodiversity pattern (Shannon index, 2.602 and 2.893, respectively), suggesting a potential contamination performed by the patient’s right hand. Conversely, sample YH1_12 displayed microbiota traces associated with the nasopharyngeal secretions and with a different biodiversity pattern (Shannon index, 2.161), suggesting a possible contact of the stethoscope. NGS found environmental bacteria of the microbiota of human biological fluids.

### Droplets and biofluids are frequently detected in fomites, representing a risk for indirect transmission of SARS-CoV-2.

**FIG 6:**
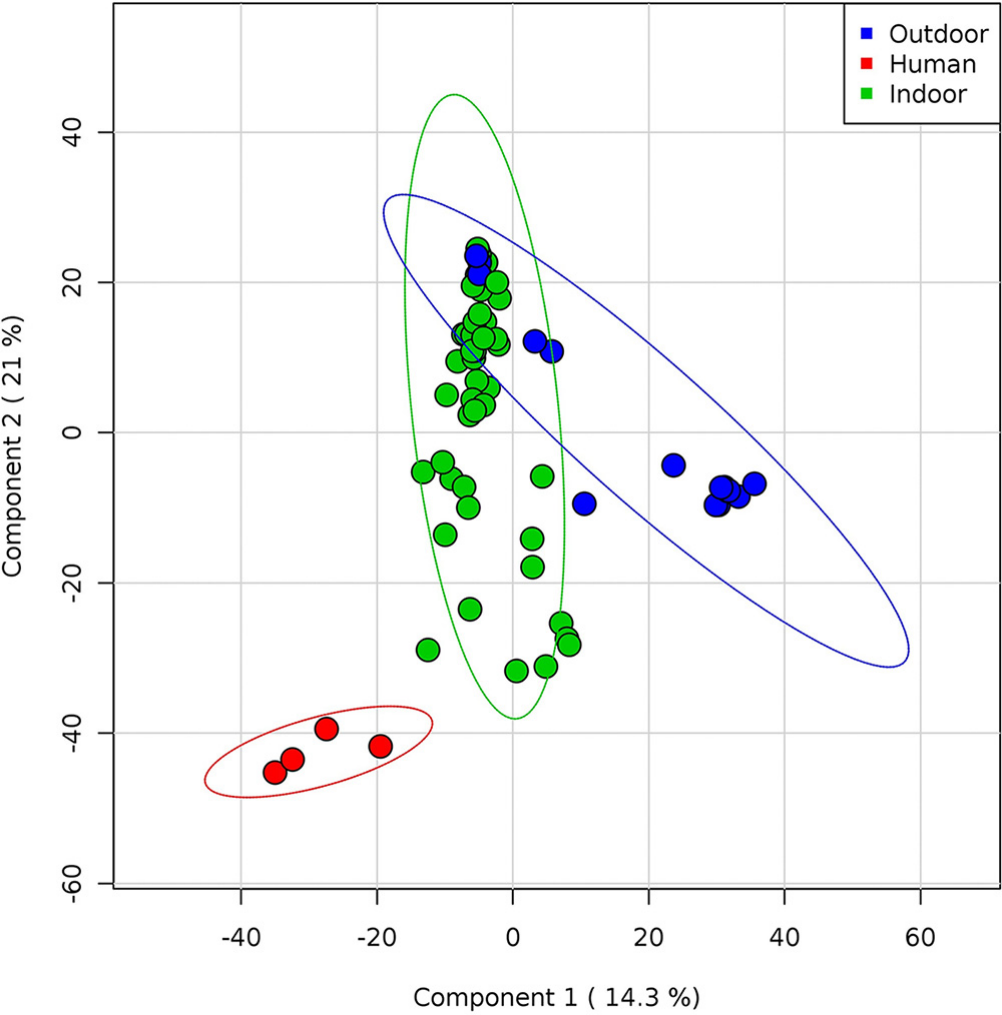
Whole microflora analysis of indoor and outdoor samples. Partial least square-discriminant analysis (PLS-DA) shows Pearson distance between different samples using phylogeny distribution based on 16S rRNA genes. Samples are colored according to the sampling point (red, human droplets; green, indoor; blue, outdoor). Components 1 and 2 explain 14.3% and 21% of the variance, respectively. Outdoor samples overlapping indoor samples are characterized by fomites, whereas the blue sample between groups is the outdoor handle of a building main entrance. All samples without a major presence of environmental microflora but characterized by a prevalence of human microbiota from droplet biofluids tend to segregate independently.

qPCR analysis of fomites allowed the identification of resident or ectopic microflora. NGS showed fomites on several surfaces exposed to anthropic contamination and inhabiting microorganisms or those from other environmental sources. Samples collected from indoor and outdoor surfaces independently grouped if compared with samples containing human biofluids (Fig. 6). Within the outdoor group, those with a higher anthropic contamination overlapped with indoor samples, far from those where the environmental component was overwhelming. Interestingly, only an outdoor sample segregated outside—and between—both groups: the external handle of the entrance of a public building (Z_04), characterized by multiple contaminations of anthropic origin overlaying the outside microflora. Other indoor samples grouped together. These findings highlight the usefulness of microbiota-related data for tracing fomites. However, if fomites can be a risk, the transmission rate is low and depends on the presence of the specific viable pathogens (48). The environmental survival of SARS-CoV-2 depends on indoor and outdoor factors, including sanitation, time of release of the biological fluid, humidity, temperature, air circulation, and sunlight (49–53). Therefore, detection of fomites should be considered an indicator of transmission risk. Monitoring droplets and biofluids by qPCR can help prevent SARS-CoV-2 transmission suggesting continuous environmental surveillance and sanitization procedures.

### Critical issues and limits of the study.

The study aim was the assessment of a novel qPCR-based approach and not a detailed risk assessment. Therefore, the comparison between sampling points or buildings cannot be performed, not only because of the small sample size but also for the random collection from areas with a different incidence of infection. Several Italian regions were recruited to avoid a geographical bias; however, the epidemiological burden can affect the inference of the results. Therefore, we suggested a strategy to assess contamination, which could point out a risk of indirect transmission of SARS-CoV-2. Interestingly, SARS-CoV-2-positive samples were collected only in Emilia-Romagna, Italy, where the incidence of infection was high during the epidemic peak (47). Nevertheless, our findings seem to confirm the key role played by hands. qPCR is faster than NGS (3 to 5 h versus 5 to 10 days) but not rapid compared with other molecular methods (33, 54). Tracing droplets and biological fluids by qPCR or NGS has a high specificity (18, 19, 55, 56). qPCR and NGS primers and probes for some bacterial genes were selected because of their feasibility and effectiveness; however, comparisons of sequences, indicators, or reaction conditions were not performed. Whole-genome sequencing would have been more informative, as well as the study of additional S16 rDNA regions (V1 and V2 together with V3, V4, V6, and V7). However, they are expensive (e.g., materials and reagents, bioinformatic analysis) and inappropriate for public health purposes on a larger scale. Finally, we used arbitrary thresholds to quantify droplets contamination based on *C_T_* values, recommending the highest sensitivity for droplet detection. A lower threshold could increase the specificity or can be helpful for monitoring transmission routes or sanitation.

### Conclusion.

Environmental monitoring of fomites can be performed by qPCR, with the identification of anthropic contaminations by detecting their microbiota component.

Droplets and biological fluids were observed in indoor environments exposed to humans and on surfaces frequently touched by hands. SARS-CoV-2 was not detectable, with the only exception of environmental areas near to a patient. Our results indirectly highlight the importance of handwashing and environmental sanitation, as well as the role of health education.

In addition to the environmental detection of SARS-CoV-2, the detection of fomites by qPCR may provide an indicator for monitoring indirect transmission of SARS-CoV-2 and other biological agents transmitted through droplets.

## MATERIALS AND METHODS

### Sampling and experimental design.

Surfaces at risk for the presence of biological fluids and the transmission of COVID-19 were sampled from different settings, both in indoor and outdoor areas. Environmental samples (*n* = 94) were collected during May-June, after the epidemic peak that occurred in Italy in March 2020. We sampled indoor surfaces from three COVID reference hospitals in three Italian regions (Parma, Emilia-Romagna; Sassari, Sardinia; Rome, Lazio), other buildings open to the public (one office, one fast food restaurant, and one church), surfaces in outdoor areas, and used handkerchiefs with nasopharyngeal secretions. All samples were tested for SARS-CoV-2 RNA, whereas anthropic contamination was assessed by searching for biological fluids from nose, mouth, and gut through detection of their microbiota traces by qPCR and/or NGS (Table 3). As reported in Table 1, the sample reading code used letters and numbers as follows: A, anthropic contamination; Z, outdoor surface; Y, indoor surface; H, hospital; C, church; O, other sampling points in office or restaurant surfaces. All A, Z, and Y samples were collected within the Rome district, while YH1, YH2, and YH3 correspond to the hospitals in Rome, Parma, and Sassari, respectively.

**TABLE 3:** Environmental sampling: data set of collected samples and testing^*a*^

Classification		No. of samples in data set (*n* = 94)	Description	Nucleic acid testing		
Surface source	Sample					
				SARS-CoV-2 (RT-qPCR)	Biofluid (qPCR)	Microbiota (NGS)
Indoor	Hospital	49	Floor (*n* = 7)	+	+	+
			Bedside table (*n* = 3)	+	+	+
			Door handle (*n* = 3)	+	+	+
			Call button (*n* = 1)	+	+	+
			Table (*n* = 1)	+	+	+
			Chair (*n* = 2)	+	+	+
			Back of the bed (*n* = 3)	+	+	+
			Side of bed (*n* = 4)	+	+	+
			Bottom of the bed (*n* = 1)	+	+	+
			Wall behind the bed head (*n* = 1)	+	+	+
			Bed sheets (*n* = 1)	+	+	+
			Pillow (*n* = 2)	+	+	+
			Stethoscope (*n* = 1)	+	+	+
			Wheelchair head (*n* = 1)	+	+	+
			Toilet board (*n* = 1)	+	+	+
			Toilet flush button (*n* = 1)	+	+	+
			Sink faucet (*n* = 1)	+	+	+
			Air circulation system (15)	+	−	−
	Public building	25	Door handle (*n* = 2)	±	+	+
			Toilet (*n* = 2)	+	+	+
			Pews (*n* = 4)	−	+	+
			Floor (*n* = 6)	±	+	+
			Toilet wall tiles (*n* = 3)	±	+	+
			Office phone (*n* = 1)	+	+	+
			Computer keyboard (*n* = 2)	+	+	+
			Air circulation system (*n* = 5)	−	+	+
Outdoor	* *	16	Handrail (*n* = 1)	+	+	+
			Grip shared e-scouter (*n* = 1)	+	+	+
			Bus stop bench (*n* = 1)	+	+	+
			Coffee dispenser button (*n* = 1)	+	+	+
			External door handle (*n* = 2)	±	+	+
			Playground (*n* = 10)	−	±	+
Human		4	Droplet biofluid (*n* = 4)	±	+	+

a Indoor surfaces were sampled from different hospitals (*n* = 3), buildings of public use (one office, one fast food restaurant, and one church), outdoor areas (*n* = 16), nose-oropharyngeal secretions (*n* = 4) and tested for the presence of SARS-CoV-2 by quantitative reverse transcription real-time PCR (RT-qPCR) and for anthropic contamination by testing the presence of microbiota traces of biological fluids (nose, saliva, Feces), by quantitative real-time PCR (qPCR) and/or next generation sequencing (NGS). For each type of test, analysis was performed in all (+), none (−), or some (±) of the collected samples.

### Sampling collection.

Surface sampling was carried out after their regular use and prolonged exposure to human presence (>4 h), following standard protocols. FLOGSwabs and CITOSSWAB were used and immediately soaked into a buffer solution of UTM-RM transport medium in a volume of 400 μl (Copan Diagnostics Inc., Murrieta, CA, USA). The nasopharyngeal secretions were collected on handkerchiefs with swabs (4N6FLOQSwabs; Copan Diagnostics Inc., Murrieta, CA, USA). All specimens were refrigerated at 4°C before being tested.

### SARS-CoV-2 detection.

All samples in UTM were heat inactivated at 56°C for 5 min to reduce the risk of accidental transmission of SARS-CoV-2 to laboratory personnel. Nucleic acids were purified and extracted using the eMag automated nucleic acid sample extraction system (bioMérieux, Marcy l’Etoile, France). Briefly, total nucleic acids were extracted from UTM using an input sample volume of 200 μl into 2,000 μl of easyMag lysis buffer using B protocol to a final eluted volume of purified nucleic acids of 50 μl. TaqPath one-step reverse transcriptase quantitative PCR (RT-qPCR) master mix (Life Technologies, Frederick, MD) and the 2019-nCoV CDC EUA kit (Integrated DNA Technologies, Coralville, IA) were used for target detection (57). Molecular detection of SARS-CoV-2 RNA was carried out by RT-qPCR, using primers and probes related to the E and N genes with a detection limit of 5.2 copies of RNA/reaction (55). Samples were analyzed in Sassari and Parma with the Allplex 2019-nCoV assay (Seegene, Seoul, South Korea) and in Rome with the Detection kit for 2019 Novel Coronavirus (2019-nCoV) RNA (PCR-Fluorescence Probing) (Daan Gene Co., Ltd., of Sun Yat-University, Guangzhou, Guandong, China) for the confirmation of the results. The Allplex 2019-nCoV assay was designed for amplifying three viral targets: the E gene (subgenus Sarbecovirus), the N, and the RdRP genes (58). The Detection kit for 2019 Novel Coronavirus (2019-nCoV) RNA (PCR-Fluorescence Probing) was designed for simultaneous identification of N and Orf1ab genes. Afterward, 5-μl portions of eluted RNA samples in a total volume of 25 μl were amplified on Bio-Rad CFX96 real-time system. In each round of extraction and amplification, positive and negative control samples (supplied by the manufacturer) were included. Laboratory controls included serial 10-fold dilutions of an internal positive sample that were analyzed in triplicate (*R*^2^ = 0.99), further supporting the reproducibility and capability to detect SARS-CoV-2 RNA up to the limit of detection of the assay. The interpretation criteria were the following: (i) positive signals detected in E, N, and RdRP ORF1ab and N genes with cycle threshold (*C_T_*) values of ≤40 were considered positive for SARS-CoV-2 RNA; (ii) positive signals in only one gene (E, N, or RdRP/Orf1ab) with *C_T_* values of ≤40 were considered inconclusive; and (iii) no fluorescent signals or over the 40th *C_T_* in RdRP/ORF1ab and N genes were considered not specific and reported as negative for SARS-CoV-2 RNA. The declared limit of detection (LoD) is 500 RNA copies/ml, following the manufacturer’s information.

### DNA extraction.

An aliquot of COPAN UTM-RM transport medium (about 300 μl) was centrifuged at 16,000 × *g* for 10 min, and the pellet was manually disaggregated with a pestle after adding glass beads (Sigma-Aldrich, St. Louis, MO) and lysed in 200 μl lysozyme solution, RNase A treated, and proteinase K digested according to the GenElute Bacterial Genomic DNA kit (Sigma-Aldrich, St. Louis, MO, USA). Finally, DNA elution was performed in 60 μl elution solution (10 mM Tris-hydrochloride and 0.5 mM ethylene diamine tetraacetic acid [pH 9.0]). For pharyngeal biofluids and fomites samples, each swab was inserted into the semipermeable NAO Baskets and broken inside at the breakpoint. Approximately 200 μl lysozyme solution (20 mg/ml lysozyme, 20 mM tris[hydroxymethyl]aminomethanehydrochloride at pH 8, 2 mM ethylenediaminetetraacetic acid, and 1.2% Triton X-100; Sigma-Aldrich, St. Louis, USA) were added into the NAO Baskets and incubated at 37°C for 30 min. Then, 20 μl proteinase K and 400 μl buffer AL were added, and the sample was centrifuged at 10,000 × *g* for 1 min, allowing the elution of the digestion solution. After incubation at 56°C for 10 min and addition of 400 μl ethanol, the washing step and DNA purification were performed in accordance with the manufacturer’s instructions. DNA elution was completed in 60 μl elution solution [10 mM tris(hydroxymethyl)aminomethane-hydrochloride and 0.5 mM ethylenediaminetetraacetic acid at pH 9.0], as previously described (32, 33).

### Analysis of mfDNA by multiplex real-time PCR and data interpretation.

Amplifications were combined in four multiplex reactions: mix skin, for the identification of Staphylococcus aureus and Staphylococcus epidermidis; mix nasopharynx for *Propionibacterium* spp. and *Corynebacterium* spp.; mix oralpharinx for Streptococcus salivarius and Streptococcus mutans; mix feces for *Enterococcus* spp. and Bacteroides vulgatus (probes were labeled FAM/VIC/HEX, with the BHQ-1 quencher). Primers for different bacterial indicators and optimized reaction conditions were already established as previously described (4, 30, 59, 60, 61). Briefly, amplifications were performed in a volume of 25 μl, of which 12.5 μl JumpStart *Taq* ReadyMix for Quantitative PCR (Sigma-Aldrich, St. Louis, MO), containing 900 nM forward and reverse primers, and 250 nM each probe. For each mix, samples were tested in triplicate. The amplifications were performed using Bio-RadCFX96 (Bio-Rad, Hercules, CA) programmed for 10 min at 95°C and 40 cycles with 1 cycle consisting of 15 s at 95°C and 1 min at 60°C. For each sample, 11 μl template reaction was amplified. The PCR output was expressed as cycle threshold (*C_T_*). Positive samples were those where ≥1 positive indicator (*C_T_* ≤ 35) was found in at least two mixes. Conversely, a microbial indicator was considered negative when it was over the *C_T_* ≥ 39 threshold. In particular, for each biological fluid, at least two bacterial markers were used, and for each indicator, the following criteria were used: +++, positive, *C_T_* < 20; ++, positive, *C_T_* of 21–30; +, positive with *C_T_* of 31 to 35; +/−, low-confidence positive with *C_T_* of 36 to 38; −, negative with *C_T_* > 39. Due to the use of recombinant and not native polymerase or Escherichia coli the criteria were modified as follows: ++, positive with *C_T_* < 20; +, positive with *C_T_* of 21 to 29; +/−, low-confidence positive with *C_T_* of 30 to 35; −, negative with *C_T_* >36; the criteria were modified in order to avoid false-positive data due to the detection of E. coli DNA in the recombinant enzyme used for amplification (62).

### 16S rDNA amplicon sequencing analysis.

Libraries for NGS were prepared according to the 16S Metagenomic Sequencing Library Preparation Guide (part 15044223 rev A; Illumina, San Diego, CA, USA). The PCR amplicons were obtained using Ba27F and Ba338R primers containing overhang adapters, as previously described (33, 63). Tagged PCR products were generated using primer pairs with unique barcodes through two-step PCR. In this strategy, target primers containing overhang adapters were used in the first PCR to amplify the target gene, that product was then used in the second PCR using primers-containing barcodes. Each amplification reaction had a total volume of 25 μl, containing 12.5 μl of KAPA HiFi Hot Start Ready Mix (Roche, Pleasanton, CA, USA), 5 μl of each primer (1 μM), and 2 μl of template DNA. Reactions were carried out on a Techne TC-PLUS thermocycler (VWR International, LLC, Radnor, PA, USA). Following amplification, 5 μl of PCR product from each reaction was used for agarose gel (1%) electrophoresis to confirm amplification. The final concentration of cleaned DNA amplicon was determined using the Qubit PicoGreen dsDNA BR assay kit (Invitrogen, Grand Island, NY, USA) and validated on a Bioanalyzer DNA 1000 chip (Agilent, Santa Clara, CA, USA). Libraries were prepared using the MiSeq reagent kit preparation guide (Illumina, San Diego, CA, USA). Raw sequence data were processed using an in-house pipeline that was built on the Galaxy platform and incorporated various software tools to evaluate the quality of the raw sequence data (FASTA/Q Information tools, Mothur). All data sets were rigorously screened to remove low-quality reads (short reads > 200 nucleotides [nt], zero-ambiguous sequences). Demultiplexing was performed to remove PhiX sequences and sort sequences; moreover, to minimize sequencing errors and ensure sequence quality, the reads were trimmed based on the sequence quality score using Btrim (an average quality score of 30 from the ends, and remove reads that are less than 200 bp after end trimming) (64). OTUs (operational taxonomic units) were clustered at a 97% similarity level, final OTUs were generated based on the clustering results, and taxonomic annotations of individual OTUs were based on representative sequences using RDP’s 16S Classifier 2.5. Observed OTUs were defined as observed species. A level of 97% sequence identity is often chosen as representative of a species and 95% for a genus (65). The sequence reads were analyzed also in the cloud environment BaseSpace through the 16S Metagenomics app (version 1.0.1; Illumina): the taxonomic database used was the Illumina-curated version (May 2013 release of the Greengenes Consortium Database) (65).

### Statistical analysis.

Relative abundances of community members were determined with rarefied data and summarized at each taxonomic level. The proportion of the microbiome at each taxonomic rank, such as phylum, order, class, family, and genus, was determined using the RDP classifier and the Greengenes Database. Alpha and beta diversity were calculated using EstimateS software at a level of 97% sequence similarity. Regarding alpha diversity, the Shannon index and equitability index at the species level were computed (66, 67). Principal-component analysis (PCA) was performed using the METAGENassist platform and R (version 3.1.3, www.R-project.org) with packages “ggplot2,” “ape,” “psych,” and “vegan” (68). Multivariate analysis, the PCA, and partial least square-discriminant analysis (PLS-DA) were performed in order to investigate the dissimilarity between groups. Feature selection was performed using PLS-DA and 10-fold cross validation to tune algorithm parameters and to check model validity. Dendrogram and clustering analysis were based on the Euclidean distance and Ward’s method.

### Data availability.

The raw sequencing data were deposited in the NCBI Sequence Read Archive under accession number PRJNA685983. The analyses were carried out using an online Galaxy server available at http://huttenhower.sph.harvard.edu/galaxy.
